# Supplementary material for: Sulforaphane Reduces Prostate Cancer Cell Growth and Proliferation In Vitro by Modulating the Cdk-Cyclin Axis and Expression of the CD44 Variants 4, 5, and 7
Source: Int J Mol Sci. 2020 Nov 18;21(22):8724. doi: 10.3390/ijms21228724 (PMC7699211; doi:10.3390/ijms21228724)

Figure S1

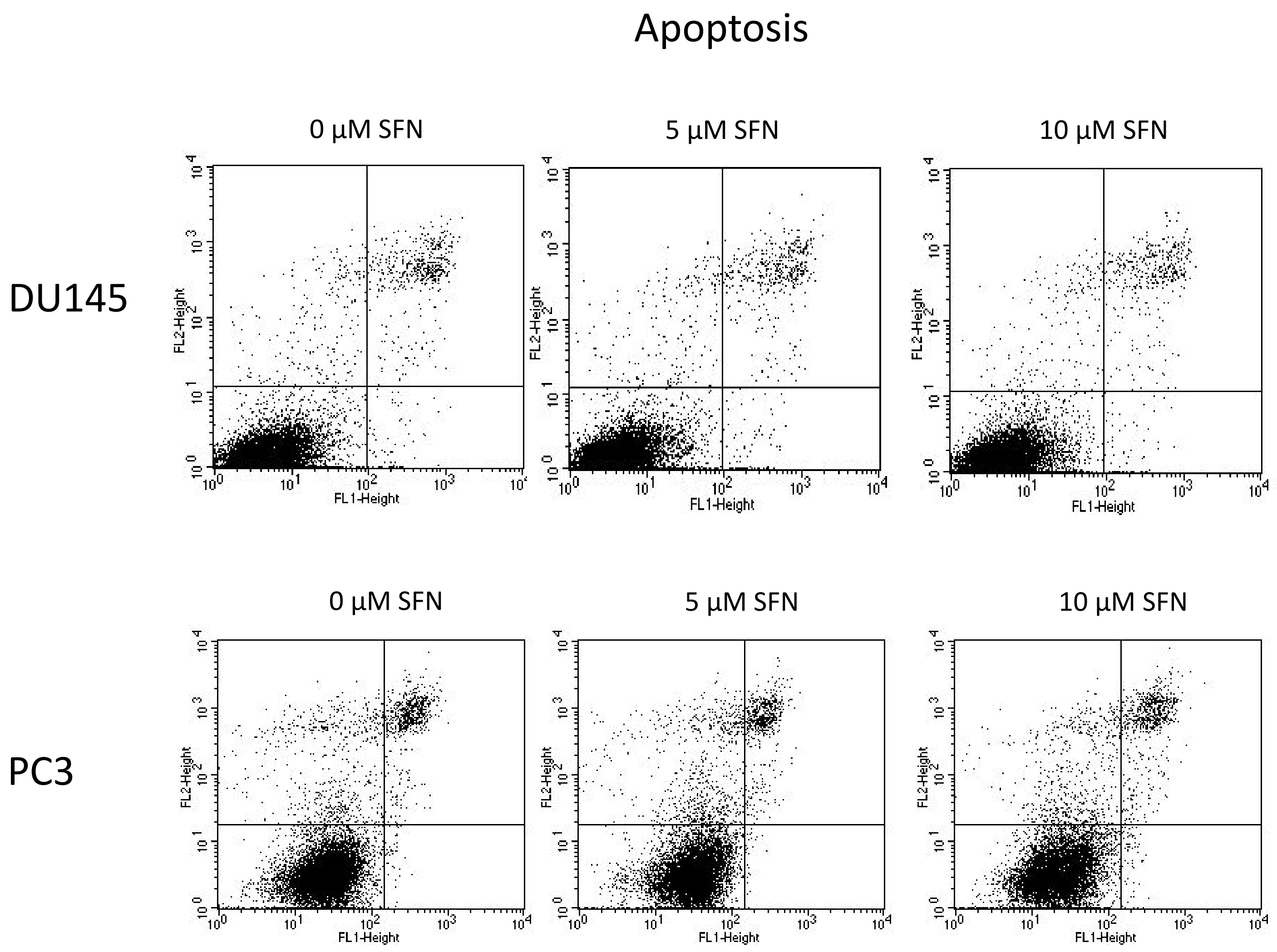
Apoptosis
0 µM SFN
5 µM SFN
10 µM SFN
DU145
PC3
FL1-Height
FL2-Height

Figure S2

Time dependent alterations of cell cycling

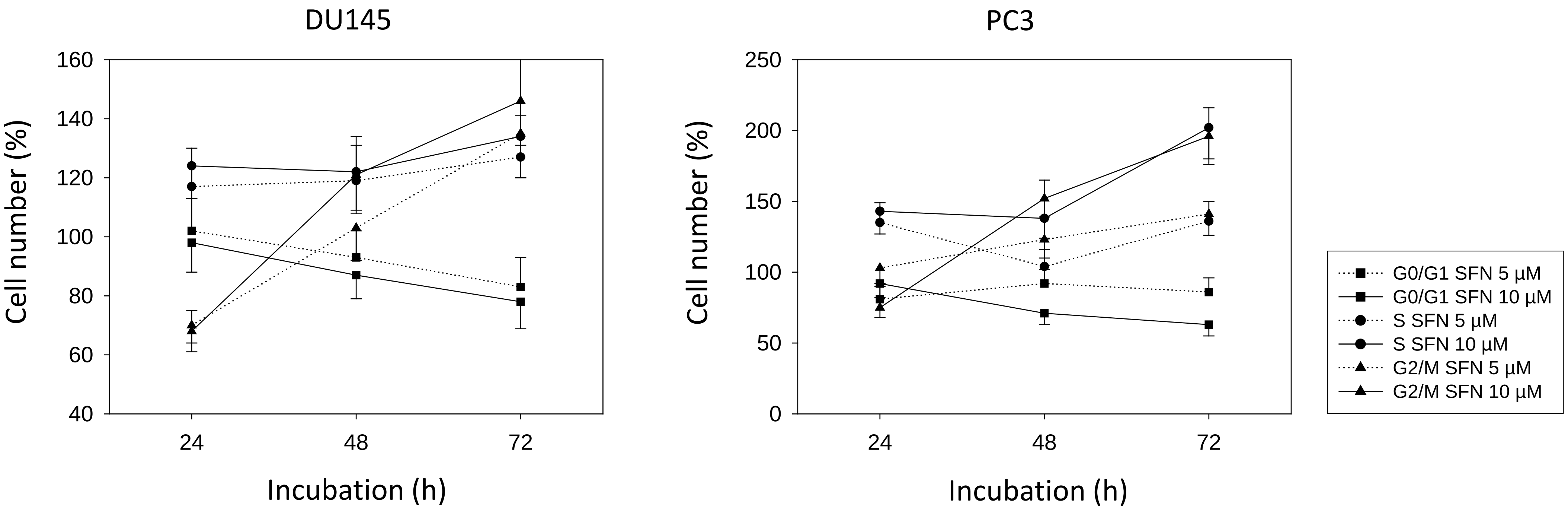

The figure depicts the number of tumor cells in G0/G1-, S- or G2/M-phase.
Cells treated with 5 versus 10 µM SFN are related to the untreated controls which were set to 100%.

Figure S3

Protein bands, corresponding to figure 4

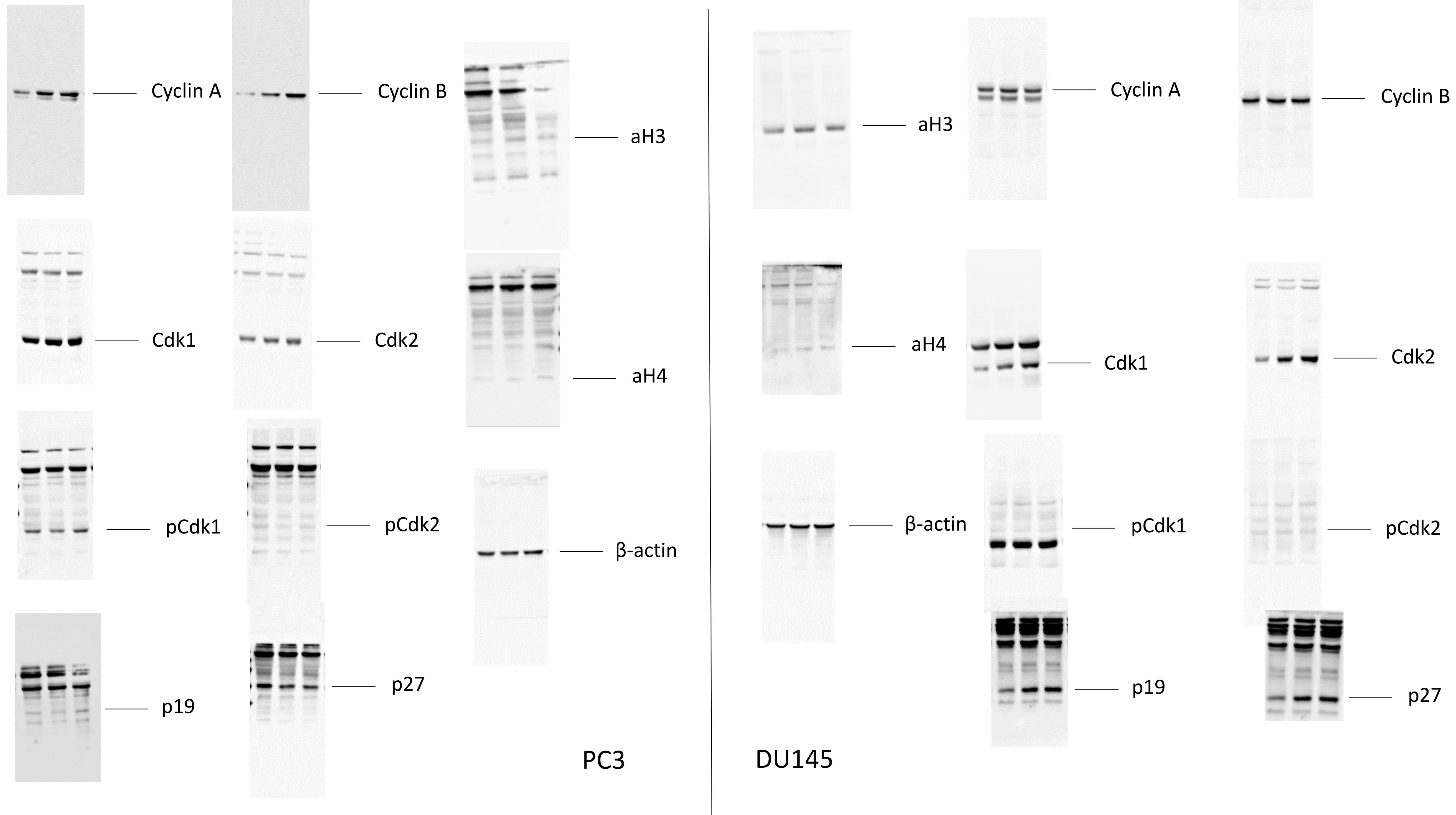

Supplement: Supplementary file 1 [file ijms-21-08724-s001.pdf]
